# Neuropilin-1 expressing melanoma cells as a model to study the aggressiveness of metastatic melanoma

**DOI:** 10.1186/1479-5876-13-S1-P6

**Published:** 2015-01-15

**Authors:** Federica Ruffini, Grazia Graziani, Lauretta Levati, Lucio Tentori, Simona Caporali, Stefania D’Atri, Pedro M Lacal

**Affiliations:** 1 Laboratory of Molecular Oncology, “Istituto Dermopatico dell’Immacolata”-IRCCS, Rome, Italy; 2 Department of Systems Medicine, University of Rome “Tor Vergata”, Rome, Italy; 3 Clinical Epidemiology Unit, “Istituto Dermopatico dell’Immacolata”-IRCCS, Rome, Italy

## Background

The molecular mechanisms associated with the acquisition of a metastatic phenotype by melanoma cells are not very well understood. Therefore, the identification of molecular determinants involved in the metastatic switch that may either cause or contribute to the aggressiveness of melanoma is of primary relevance.

We had previously identified neuropilin-1 (NRP-1), a co-receptor of the vascular endothelial growth factor-A (VEGF-A), as an important determinant of melanoma aggressiveness, in clones of the human melanoma cell line M14, expressing or not NRP-1 [1,2]. We demonstrated that even though the simultaneous presence of both VEGFR-2 and NRP-1 potentiates VEGF-A secretion and the aggressiveness of melanoma cells, NRP-1 is by itself able to promote cell invasion [1].

During melanoma progression, tumour cells show increased adhesiveness to the vascular wall, invade the extracellular matrix (ECM) and frequently form functional channels similar to vascular vessels (vasculogenic mimicry) [3]. In the present study we analysed the mechanisms responsible for the aggressive phenotype of NRP-1 expressing melanoma cells.

## Materials and methods

Melanoma aggressiveness was evaluated *in vitro* as cell ability to migrate through an ECM layer in Boyden chambers and to form tubule-like structures on matrigel gels. Pre-incubation of the cells with specific blocking antibodies allowed the identification of specific integrins and other molecules relevant to these processes. The results obtained by anti-integrin antibodies, showing the involvement of αvβ5 integrin in the aggressiveness of melanoma cells expressing NRP-1, were confirmed by *ITGB5* gene silencing and by the use of cilengitide, a potent inhibitor of αν integrins activation.

## Results

The expression of αvβ5 integrin was found to be twice higher in NRP-1 expressing melanoma cells than in the low-invasive NRP-1 negative control. Its blockage resulted in a significant decrease of the ability of NRP-1 expressing cells to invade ECM and to form tubule-like structures on matrigel. Cilengitide and *ITGB5* silencing reduced ECM invasion and vasculogenic mimicry. Moreover, cilengitide down-modulated the secretion of VEGF-A and metalloproteinase-9 (MMP-9). Finally, melanoma cells expressing NRP1, but lacking other VEGF-A or PlGF receptors (VEGFR-1 and VEGFR-2), specifically responded to PlGF in a chemotactic assay.

## Conclusions

In conclusion, we identified novel mechanisms that modulate melanoma aggressiveness involving NRP-1, ανβ5 integrin and PlGF, which might be considered as new targets of therapeutic strategies to inhibit the metastatic disease.
